# Inhibition of mutant KRAS-driven overexpression of ARF6 and MYC by an eIF4A inhibitor drug improves the effects of anti-PD-1 immunotherapy for pancreatic cancer

**DOI:** 10.1186/s12964-021-00733-y

**Published:** 2021-05-17

**Authors:** Ari Hashimoto, Haruka Handa, Soichiro Hata, Akio Tsutaho, Takao Yoshida, Satoshi Hirano, Shigeru Hashimoto, Hisataka Sabe

**Affiliations:** 1 Department of Molecular Biology, Hokkaido University Faculty of Medicine, N15W7 Kita-ku, Sapporo, Hokkaido 060-8638 Japan; 2 Department of Gastroenterological Surgery II, Faculty of Medicine, Hokkaido University, N15W7 Kita-ku, Sapporo, Hokkaido 060-8638 Japan; 3 Research Center of Oncology, Ono Pharmaceutical Co., Ltd., 3-1-1 Sakurai, Shimaoto-cho, Mishima-gun, Osaka 618-8585 Japan; 4 Present Address: Laboratory of Immune Regulation, Immunology Frontier Research Center, Osaka University, Osaka, 565-0871 Japan

**Keywords:** ARF6, AMAP1, eIF4A inhibitor, Anti-PD-1 therapy

## Abstract

Many clinical trials are being conducted to clarify effective combinations of various drugs for immune checkpoint blockade (ICB) therapy. However, although extensive studies from multiple aspects have been conducted regarding treatments for pancreatic ductal adenocarcinoma (PDAC), there are still no effective ICB-based therapies or biomarkers for this cancer type. A series of our studies have identified that the small GTPase ARF6 and its downstream effector AMAP1 (also called ASAP1/DDEF1) are often overexpressed in different cancers, including PDAC, and closely correlate with poor patient survival. Mechanistically, the ARF6-AMAP1 pathway drives cancer cell invasion and immune evasion, via upregulating β1-integrins and PD-L1, and downregulating E-cadherin, upon ARF6 activation by external ligands. Moreover, the ARF6-AMAP1 pathway enhances the fibrosis caused by PDAC, which is another barrier for ICB therapies. *KRAS* mutations are prevalent in PDACs. We have shown previously that oncogenic *KRAS* mutations are the major cause of the aberrant overexpression of ARF6 and AMAP1, in which KRAS signaling enhances eukaryotic initiation factor 4A (eIF4A)-dependent *ARF6* mRNA translation and eIF4E-dependent *AMAP1* mRNA translation. MYC overexpression is also a key pathway in driving cancer malignancy. *MYC* mRNA is also known to be under the control of eIF4A, and the eIF4A inhibitor silvestrol suppresses MYC and ARF6 expression. Using a KPC mouse model of human PDAC (*LSL-Kras(G12D/+); LSL-Trp53(R172H/*+*)); Pdx-1-Cre*), we here demonstrate that inhibition of the ARF6-AMAP1 pathway by shRNAs in cancer cells results in therapeutic synergy with an anti-PD-1 antibody in vivo; and furthermore, that silvestrol improves the efficacy of anti-PD-1 therapy, whereas silvestrol on its own promotes tumor growth in vivo. ARF6 and MYC are both essential for normal cell functions. We demonstrate that silvestrol substantially mitigates the overexpression of ARF6 and MYC in KRAS-mutated cells, whereas the suppression is moderate in KRAS-intact cells. We propose that targeting eIF4A, as well as mutant KRAS, provides novel methods to improve the efficacy of anti-PD-1 and associated ICB therapies against PDACs, in which ARF6 and AMAP1 overexpression, as well as *KRAS* mutations of cancer cells are biomarkers to identify patients with drug-susceptible disease. The same may be applicable to other cancers with *KRAS* mutations.

**Video abstract**

**Supplementary Information:**

The online version contains supplementary material available at 10.1186/s12964-021-00733-y.

Although many clinical trials are being conducted to clarify effective combinations of drugs for immune checkpoint blockade (ICB) therapies, the characteristics of patients in whom such combination therapies will be effective remain unclear. Pancreatic ductal adenocarcinomas (PDACs) are refractory to the currently available immune checkpoint blockade (ICB) therapies, and the five-year overall survival rate remains at no more than 10% [1]. A double mutation in *KRAS* and *TP53* is a hallmark of PDAC [2], although it seldomly occurs in other types of cancers unless they become highly malignant. Mutations in the cell cycle suppressor *CDKN2A* and the tumor suppressor *SMAD4/DPC4* also frequently occur in PDACs, and these mutations are thought to further facilitate cancer cell proliferation, primarily driven by *KRAS/TP53* mutations [3]. On the other hand, locally advanced malignancy (i.e., tumor invasion into the surrounding tissues) already at the time of the initial diagnosis is another hallmark of PDACs, and is closely associated with their refractory nature [4]. Metastases are also frequently observed at the initial diagnosis of PDACs [4].

ARF6 is a small GTPase that is ubiquitously expressed in various types of normal cells, and is primarily involved in the recycling of a variety of plasma membrane components [5, 6]. A series of our studies have identified that ARF6 and one of its downstream effectors, namely, AMAP1 (also called ASAP1 and DDEF1), are often overexpressed in many different cancer cells, including PDAC, breast cancer, clear cell renal cell carcinoma, and lung adenocarcinoma, and that this overexpression statistically correlates with poor patient survival [7–12]. The ARF6-AMAP1 pathway regulates intracellular dynamics of the β1-integrins, E-cadherin, and PD-L1, and hence modulates cell adhesion to the stroma, as well as to other cells, including immune cells, to promote invasion, metastasis, and immune evasion [12–14]. The ARF6-AMAP1 pathway also increases cell-surface levels of the β1-integrins and PD-L1, and also the fibrosis caused by PDAC, which is another barrier for immunotherapy [12, 13, 15]. This pathway furthermore regulates the intracellular distribution of mitochondria, and is hence indispensable for avoiding mitochondria-based oxidative catastrophe during cell invasion into narrow paths [16]. In this pathway, ARF6 is activated by external ligands, such as those for various receptor tyrosine kinases and G-protein-coupled receptors [8, 11]. Moreover, mevalonate pathway activity is essential for the activation of ARF6 by external ligands [17]. It has also been reported by other research groups that ARF6 is involved in maintenance of the Warburg effect, to meet the unusual nutrient/energy demands of cancer cells [18], in the trafficking of pre-miRNA complexes to sites of microvesicle biogenesis [19], and their RhoB-mediated subcellular targeting to endosomes, and for the various biological functions of cancer cells [20]. AMAP1 may also directly bind to actin filaments to bundle them to be an integral part of the actin stress fiber organization [21]. Moreover, intriguingly, the Analytical Multi-scale Identification of Recurring Events (ADMIRE) algorithm has identified that the *AMAP1* gene is frequently amplified in triple-negative breast cancer, and acts as a key driver of cancer progression and recurrence, in which AMAP1 appears to negatively regulate cell death pathways [22].

We recently clarified that the *KRAS*/*TP53* double mutation activates the ARF6-AMAP1 pathway, i.e., oncogenic *KRAS* mutations promote the eukaryotic initiation factor 4A (eIF4A)- and eIF4E-dependent mRNA translation processes, leading to overexpression of the ARF6 protein and AMAP1 protein, respectively. In addition, oncogenic *TP53* mutations have been shown to facilitate processes activating ARF6 by external ligands, via their known functions in promoting the expression of platelet-derived growth factor receptor and several enzymes involved in the mevalonate pathway [12]. Consistently, tumor cells arising in the representative mouse model of human PDAC, namely, KPC mice (*LSL-Kras(G12D/*+*); LSL-Trp53(R172H/*+*); Pdx-1-Cre*), express Arf6 and Amap1 at high levels, and use them to drive processes involved in malignancy, including invasion, immune evasion, and fibrosis in vivo [12, 15].

eIF4A is an RNA helicase that is necessary to unwind secondary structures of mRNA, including G-quadruplex (G4) structures, to initiate the elongation stage of translation [23], and is inhibited by silvestrol [24, 25]. Human and mouse ARF6/Arf6 mRNAs contain G4 structures [12], and we previously demonstrated that silvestrol substantially reduces ARF6/Arf6 protein levels in KRAS-mutated PDAC cells [12]. On the other hand, other genes, including *MYC*, also contain G4 structures, and silvestrol was shown to suppress MYC expression [23]. It was also shown that mutant KRAS promotes MYC expression, although whether this augmentation is mediated by eIF4A has not been confirmed [26]. MYC is a master regulator of a number of cellular processes and activities, including mitochondrial biogenesis and functions [27, 28]. On the other hand, overexpression of MYC is well known to be a key molecule in driving the proliferation and malignancy of different types of cancers, including PDAC [26, 29]. Likewise, ARF6 is essential not only for driving malignancy, but also for the function of normal cells, as mentioned above. Thus, the suppression of eIF4A activity is expected to be detrimental to normal cells, including immune cells.

In this study, we aimed to clarify whether inhibition of the ARF6-AMAP1 pathway in cancer cells improves the anti-tumor effects of an anti-PD-1 antibody (Ab) in vivo. For this purpose, we used the KPC mouse model of human PDAC, in which KPC cancer cells are injected into their syngeneic immunocompetent C57BL/6 mice. We previously showed that the pretreatment of KPC cells with *shAmap1* significantly suppresses the growth of KPC cells in syngeneic immunocompetent mice, but not in immune-deficient mice [12]. We here found that the pretreatment of KPC cells with *shAmap1* (*shAmap1* #1 and #2) results in significant synergy with the anti-PD-1 Ab in suppressing tumor growth in vivo, in which the Ab was administered to mice on days 10, 14, and 17 after tumor cell injection (Fig. 1a). Then, we tested the effects of silvestrol, and first found that the administration of silvestrol without the anti-PD-1 Ab promoted tumor growth (Fig. 1b). This was an expected adverse effect of silvestrol, likely owing at least partly to the silvestrol-mediated reduction in ARF6 and MYC levels in a variety of normal cells of C57BL/6 mice. However, we next found that silvestrol demonstrates robust synergy with the anti-PD-1 Ab in intact KPC cells, as seen with *shAmap1* pretreatment (Fig. 1b); while silvestrol and the anti-PD-1 Ab more severely suppressed the growth of *shAmap1* pretreated cells than intact KPC cells (Fig. 1b). A previous report suggested that silvestrol may substantially suppress MYC expression [23]. However, we demonstrated that although silvestrol substantially suppresses overexpressed ARF6 and MYC in KRAS-mutated cells, the suppression of these proteins by silvestrol was moderate in KRAS-intact cells (Fig. 1c).

**Fig. 1:**
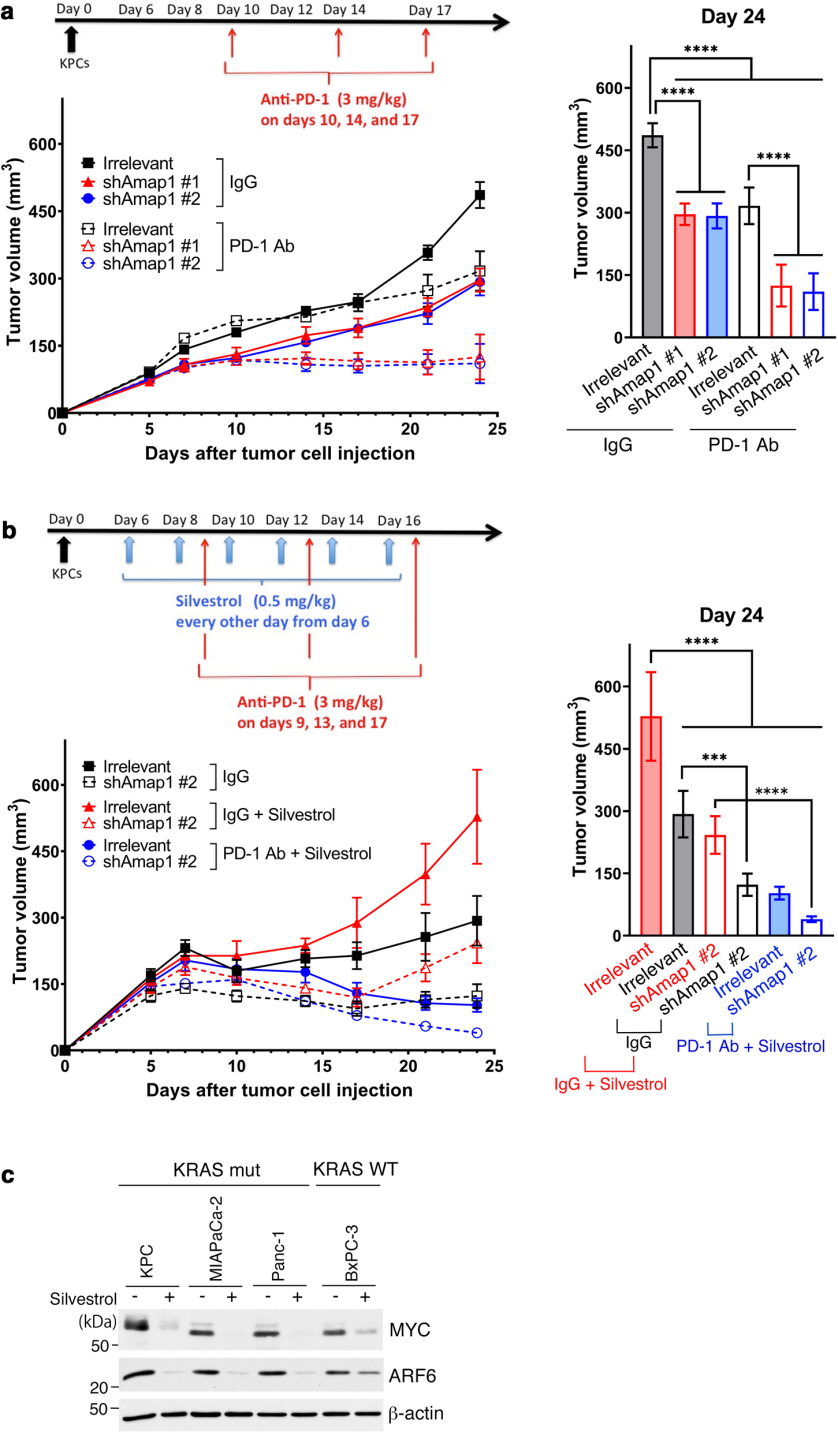
Targeting the Arf6-Amap1 pathway by *shAmap1* or silvestrol leads to therapeutic synergy with anti-PD-1 ICB. **a** On day 0, C57BL/6 mice (8 to 10-week old females, CLEA Japan) were injected subcutaneously with 2 × 10^6^ KPC cells, in which *Amap1* was silenced by shRNAs (*shAmap1* #1 and #2) or that were treated with an irrelevant shRNA (Irr), as described previously [12]. On days 10, 14, and 17, mice were injected intraperitoneally with 3 mg/kg of anti-PD-1 Ab or control IgG according to European Medicines Agency (https://www.ema.europa.eu/en/documents/assessment-report/nivolumab-bms-epar-public-assessment-report_en.pdf). Tumor sizes were measured every 2 to 4 days, starting on day 5. Tumor sizes measured on day 24 are shown in the right panel. Error bars represent the mean ± s.e.m. ****P < 0.0001. **b** C57BL/6 mice, inoculated subcutaneously with control KPC cells (Irr) or *Amap1*-silenced KPC cells (*shAmap1* #2) as in **a**, were treated with 3 mg/kg of anti-PD-1 Ab, control IgG, or 0.5 mg/kg of silvestrol, as indicated in the timeline. Tumor sizes measured on day 24 are shown in the right panel. Error bars represent the mean ± s.e.m. ***P < 0.001, ****P < 0.0001. **c** Western blot demonstrating the suppression of MYC and ARF6 levels by silvestrol in PDAC cells. *β*-Actin was used as a control

These results indicated that although suppression of the Arf6-Amap1 pathway on its own mitigates the immune evasive properties of KPC tumor cells to some extent, as we have shown previously [12], inhibition of this pathway results in therapeutic synergy with the anti-PD-1 Ab. Consistent with this notion, although silvestrol on its own exerted protumor effects, its combination with the anti-PD-1 Ab resulted in anti-tumor effects that were much stronger than those observed with the anti-PD-1 Ab alone. Our results moreover suggest that it might be possible to determine dosages of the eIF4A inhibitor(s) that are low enough to mitigate mutant KRAS-driven overexpression of ARF6 and MYC in cancer cells, with minimal effects on normal cells. On the other hand, silvestrol is not yet applicable to humans. A number of preclinical studies are ongoing with regard to silvestrol and its associated eIF4A inhibitors; and it might hence be possible to improve these drugs to be applicable to human tumors, as in the case of the development of temsirolimus and everolimus from the original mTOR inhibitor rapamycin [30]. It should also be noted that mutant RAS is now directly druggable [31]. Therefore, targeting mutant KRAS might also be effective against cancer cells with a highly active ARF6-AMAP1 pathway, when combined with specific ICB therapies.

## Conclusions

A series of our studies have shown that the ARF6-AMAP1 pathway, when overexpressed in cancer cells, provides different molecular targets that block this pathway, including certain types of receptor tyrosine kinase and G-protein-coupled receptors, the mevalonate pathway, mTORC1, and eIF4A. Inhibition of this pathway in cancer cells not only mitigates their invasion, metastasis, and immune evasion, but also makes cancer cells prone to death by enhancing their mitochondria-based oxidative catastrophe. Our recent analyses have furthermore demonstrated that ARF6-AMAP1 pathway activity is crucially involved in determining the properties and numbers of tumor-infiltrating leukocytes (our unpublished results). Together with the results shown herein, we hence propose that pharmacological inhibition of the ARF6-AMAP1 pathway will be useful for improving the effects of anti-PD-1 therapies and other ICB therapies, including anti-PD-L1 therapy. Furthermore, high expression levels of ARF6 and/or AMAP1, as well as the *KRAS* mutation, may act as biomarkers to identify patients in whom these treatments are effective.

## Data Availability

All data that were obtained in this study are included in this article.

## Abbreviations

Ab: Antibody
eIF4A/4E: eukaryotic initiation factor 4A/4E
ICB: Immune checkpoint blockade
PDAC: Pancreatic ductal adenocarcinoma
s.e.m: Standard error of the mean
